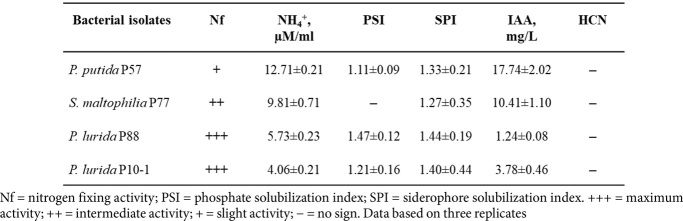# Isolation and Characterization of Multi-Trait Plant Growth-Promoting Endophytic Bacteria from Scots Pine Tissues

**DOI:** 10.4014/jmb.2408.08056

**Published:** 2024-11-22

**Authors:** Yuliia I. Shalovylo, Yurii M. Yusypovych, Oleh Y. Kit, Valentina A. Kovaleva

**Affiliations:** 1 Ukrainian National Forestry University, 103 Gen Chuprynky Str., Lviv 79057, Ukraine; 2 Institute of Cell Biology, National Academy of Sciences of Ukraine, 14/16 Drahomanova Str., Lviv 79005, Ukraine; 3 Sudova Vyshnya Lyceum Named after Tadei Dmytrasevych, Lviv Region, Ukraine

**Keywords:** Endophytic bacteria, *Pinus sylvestris* L., *Pseudomonas*, *Stenotrophomonas*, growth-promoting potential, seed inoculation

## Abstract

Scots pine (*Pinus sylvestris*) is a globally significant tree species with considerable economic importance in forestry. A major challenge in afforestation, particularly in stressful environments, is growing seedlings with high viability and stress resistance. Recent studies suggest that *Pseudomonas* strains can alleviate stress and promote growth in crops, though limited evidence exists for trees. This study aimed to assess the plant growth-promoting (PGP) properties of *Pseudomonas* strains isolated from Scots pine stems using *in vitro* assays, and to evaluate their potential as bioinoculants through a two-year long field trial. From over sixty bacterial isolates originating from Scots pine stem tissues, only four were selected as being similar to *Pseudomonas* bacteria. Through 16S rRNA gene sequencing, the isolates were identified as *Pseudomonas putida* P57, *Pseudomonas lurida* P88 and 10-1, and *Stenotrophomonas maltophilia* P77. All isolates inhibited fungal pathogens *Botrytis cinerea* and *Fusarium sporotrichiella*, and exhibited PGP activities including nitrogen fixation and production of IAA (1.24-17.74 mg/l), ammonia (4.06-12.71 μM/ml), and siderophores, with the highest value of 1.44 ± 0.19 for the *P. lurida* P88 strain. Additionally, the *Pseudomonas* strains demonstrated phosphate solubilization capacity. We revealed that bioinoculation with strains P57 and P88 enhanced field germination of seeds by 35-45% and increased aerial biomass of two-year-old seedlings by 80-140%. Both strains adhered to seed surface and colonized roots and stems at levels of 2.4-3.2 log CFU/g fresh tissue up to two years post-inoculation. These findings highlight the potential of these bacterial strains as effective bioinoculants for improving Scots pine seedling growth under natural conditions.

## Introduction

Plants are closely associated with a myriad of microorganisms, including protists, fungi, and bacteria, which together form their microbiome. The vast majority of plants on Earth are colonized by endophytes [1], and a plant without endophytes is a rare exception in nature [2]. Endophytic bacteria benefit plants by promoting growth, suppressing pathogens, enhancing stress resistance, and boosting immunity [3]. Since they share ecological niches with phytopathogens, endophytes can help protect plants from harmful microbes [4].

Bacterial endophytes similar to plant growth-promoting rizobacteria can directly promote plant growth through the production of phytohormones (indole-3-acetic acid (IAA), gibberellic acid, etc.), phosphate solubilization, nitrogen fixation, siderophore, hydrogen cyanide (HCN), and ammonia production [5-8]. They also contribute indirectly by suppressing disease by producing antibiotics and secondary metabolites, inducing systemic resistance, protecting against pests, and performing metal bioremediation to aid growth in contaminated soils [9-11].

Endophytic bacteria have been isolated from various plant parts, including roots, stems, leaves, and seeds in multiple plant species [5], with genera such as *Bacillus*, *Pseudomonas*, *Burkholderia*, and *Microbacterium* being common, among which, *Bacillus* and *Pseudomonas* are the predominant genera [12-14]. Due to the significant benefits of endophytic bacteria for plant growth and development, many studies focus on understanding the species composition of endophytic microbiomes in agriculturally important plants and characterizing these bacterial isolates. Research indicates that endophytic bacteria possess broad plant growth-promoting (PGP) potential and can be utilized as bioinoculants to support sustainable agriculture [13]. Evidence also shows that bacterial strains within the same species can display varying metabolic and functional capabilities due to genomic differences [15]. This genomic and metabolic variability contributes to the ecological differentiation among strains [16]. Therefore, the search for new strains of endophytic bacteria in various ecological niches that have beneficial effects on plants and are capable of forming compatible associations with all agronomically important plants opens up prospects for creating effective microbial preparations for adaptive crop production [17].

Most of our knowledge about bacterial endophytic microbiomes comes from studies on crop plants and the model species *Arabidopsis thaliana* [18-20], while much less is known about their roles in long-lived forest plants, particularly conifers. Many conifer species lack cultured endophytic microbes, limiting our understanding of how these bacteria help trees survive in harsh conditions, such as poor soils and high elevations.

Several studies examining the endophyte microbiome in conifer trees have have identified various bacterial genera within the pine holobiont. For instance, *Mycobacterium*, *Methylobacterium*, and *Pseudomonas* species have been found in the meristematic tissues of Scots pine buds [21]. Additionally, *Bacillus*, *Pseudomonas*, *Micrococcus*, *Serratia*, *Enterobacter*, *Pantoea*, *Staphylococcus*, and *Microbacterium* have been isolated from Scots pine roots [22]. Other studies identified *Paenibacillus* and *Bacillus* in the roots of *P. sylvestris* [23] and *Pinus contorta* [24], while N_2_-fixing *Acetobacteraceae* members were part of the needle endophytic microbiome of *Pinus flexilis* and *Picea engelmannii* [25].

Scots pine is a widespread species of pine that grows naturally over a large area of Eurasia. It is a pioneer species, frost- and drought-tolerant, capable of growing in very poor soils, and can be found in many different environments [26]. However, climate fluctuations negatively affect its photosynthesis, growth [27], and resistance to pests and fungal pathogens [28, 29]. Recent research shows that plant microbiomes can help mitigate these impacts by enhancing nutrition, growth, defense against pathogens, and tolerance to abiotic stresses [30].

Among plant beneficial endophytic bacteria, *Pseudomonas* species are noted for their potential to alleviate environmental stresses in plants [31, 32]. Numerous reports have demonstrated the effectiveness of inoculating crops with *Pseudomonas* bacteria in alleviating heat and salt stress, drought, and flooding, *i.e.*, stresses closely linked to climate change [33-36]. While inoculating *Pinus contorta* with endophytes has shown benefits for seedling growth in poor soil, similar studies for pine are scarce [37, 38].

Scots pine is a long-lived plant that experiences significant fluctuations in climatic conditions during its ontogeny and inhabits areas with poor soils. Therefore, the search for strains of endophytic bacteria from Scots pine with PGP abilities is promising for sustaining the growth of pine trees under nutrient-poor edaphic conditions in the context of global climate change.

This research aimed to investigate the PGP capabilities of *Pseudomonas* strains isolated from Scots pine stems through *in vitro* assays and determine their suitability as bioinoculants during a two-year field study.The objectives of our study were to: i) isolate *Pseudomonas* bacteria from Scots pine tissues; ii) screen selected isolates *in vitro* for PGP activities including nitrogen fixation, phosphorus solubilization, and production of ammonia, siderophore, IAA, and hydrogen cyanide; iii) evaluate the activity of isolates against fungal pathogens *B. cinerea* and *F. sporotrichiella*, and iv) assess the plant growth-promoting and colonization ability of these bacterial strains *in planta* by inoculating them into their natural host (Scots pine) in both laboratory tests and a 2-year growth trial under field conditions. Since previous studies on the PGP potential of endophytic bacteria for conifer species were conducted under controlled conditions (greenhouse) or in short-term field trials, our two-year field trial the investigating the beneficial effects of *Pseudomonas* endophytes on Scots pine seedlings under natural field conditions is pioneering.

## Materials and Methods

### Biological Material

For endophytic bacteria isolation, samples were collected from healthy Scots pine trees located at Sudova Vyshnya forestry (49°48'19.0''N 23°22'27.9''E) in the Lviv region of Ukraine. A sterilized knife was used to cut pine tissues from under the bark of the trunk, which were then placed in plastic bags. The samples were transported to the laboratory in an ice cooler box and used for bacterial isolation within 24 h. Scots pine seeds were gathered from trees in the same region in February and stored at +4°C in the dark.

The fungal cultures of *Fusarium sporotrichiella* Bilai UKM F-55297 and *Botrytis cinerea* (Persoon) Fries UKM F-2246 were obtained from the Zabolotny Institute of Microbiology and Virology (Ukraine). The fungi were maintained on potato-dextrose agar (PDA).

### Endophytic Bacteria Isolation

Pieces of Scots pine wood were thoroughly washed with tap water. They were then surface-sterilized by dipping them into 70% (v/v) ethanol for 5 min, followed by treatment with a 5% (v/v) aqueous solution of sodium hypochlorite for 5 min. Finally, the wood pieces were rinsed twice with autoclaved distilled water [22]. The success of sterilization was assessed by plating an aliquot of the final wash onto LB agar, incubating it at 30°C for 24-72 h, and checking for microbial growth on the surface of the nutrient medium.

The sterilized wood pieces were cut into small fragments under aseptic conditions and placed on the surface of LB-agar Petri dishes. The plates were incubated at 30°C and checked daily for bacterial growth. The bacterial colonies that appeared were then picked up and re-plated on fresh LB agar medium to obtain pure cultures. The bacterial strains were stored as 30% glycerol stocks at −20°C for further analysis.

The isolated strains were grouped according to colony morphology, cell shape, growth rate, and Gram reaction. Rod-shaped, motile, gram-negative bacteria were selected for further identification. The biochemical characteristics of the selected isolates were tested using standard methods [39]. To screen for antibiotic susceptibility, the isolates were grown on nutrient agar with a range of antibiotics, including ampicillin (100 μg/ml), streptomycin (50 μg/ml), tetracycline (5 μg/ml), kanamycin (15 μg/ml), rifampicin (10 μg/ml), and nalidixic acid (20 μg/ml). Four bacterial strains were selected for molecular identification.

### Molecular Identification of Endophytic Bacteria

The bacterial isolates P57, P77, P88, and P10-1 were grown on LB agar for 72 h at 30°C. Bacterial genomic DNA was isolated using a QIAamp DNA Kit (Qiagen). Amplification of the 16S rRNA gene was performed using the universal primer pairs 8F (5’-AGAGTTTGATYMTGGCTCAG-3’) and 1510R (5’-TACGGYTACCTTGTTACGACTT-3’)[40, 41]. The PCR products were sent to Explogen LLC (EXG, Lviv, Ukraine) for Sanger sequencing.

To identify the closest taxa, the 16S rRNA sequences were aligned with those from the National Center for Biotechnology Information nucleotide nr/nt (non-redundant nucleotide) database using the BLASTN algorithm (https://blast.ncbi.nlm.nih.gov/Blast.cgi) and submitted to the GenBank database to obtain accession numbers. A phylogenetic tree was constructed using the maximum likelihood method with MEGA version 11 software [42], specifically designed for the 16S rRNA sequences, to determine the phylogenetic position of the isolates.

### PGP Properties Screening

**Nitrogen Fixation Capability**

Molecular nitrogen fixation was tested on nitrogen-free medium, such as Ashby-sucrose agar [43] and Nfb (nitrogen-free bromothymol blue) semi-solid medium [44, 45]. The Ashby medium was inoculated by streaking from bacterial cultures, and incubating at 30°C for 5 days, until bacterial growth was observed. The cultures were then successively passaged twice to confirm strain growth. Any growth on the medium indicated the bacteria's ability to fix nitrogen.

The NFb-medium was used for visual detection of nitrogen-fixing activity in bacterial strains. The test involved growing 1 ml of bacterial suspension (10^8^ CFU/ml) in 9 ml of Nfb semi-solid media. Nitrogen-fixing ability of isolates P57, P77, P88, and P10-1 was indicated by a color change, turning blue or dark blue after 48 h of incubation.

### Phosphate Solubilization

The phosphate-solubilizing abilities of the tested isolates were measured on Pikovskaya agar (PVK) medium [46]. Briefly, 5 μl of a fresh suspension of each isolate (10^8^ CFU/ml) was spotted onto a PVK agar plate and incubated at 30°C. The phosphate solubilization potential of the isolate was indicated by the formation of a halo zone surrounding the sites of bacterial colonies. The phosphate solubilization index (PSI) was calculated after 5 days of incubation as follows [47]:

PSI = (colony diameter + halo zone diameter) / colony diameter

### Indole Acetic Acid (IAA) Production

The *in vitro* production of IAA by each isolate was determined colorimetrically following the method of Gordon and Weber (1951) [48]. The bacteria were grown in LB medium supplemented with 5 mM L-tryptophan at 30°C for 72 h. The supernatants were then collected by centrifugation at 5,500 rpm for 5 min. One milliliter of the culture filtrate was allowed to react with 4 ml of Salkowski reagent (1 ml 0.5 M FeCl_3_, 30 ml 98% H_2_SO_4_, 50 ml distilled water) at room temperature in the dark for 20 min [49, 50]. The optical density (OD) of the solution was measured at 535 nm using a UV-Vis spectrophotometer ULAB 102-UV (China). The concentration of IAA produced by each isolate was then determined using a standard curve according to the following equation:

Y=0.0208x+0.1196, R^2^=0.9648.

### Ammonia Production Assay

The ammonia production was revealed using a spectrophotometric quantification assay with addition of Nessler’s reagent [51]. The bacterial strains were pre-grown in LB medium for 24 h at 30°C. Half a milliliter of the bacterial supernatant was mixed with 1 ml of Nessler’s reagent in test tubes previously washed with ultrapure water. The reaction mixture was incubated at room temperature (25°C) for 10 min, then diluted 6x to a final volume of 9 ml [52]. The absorbance was measured at 450 nm using a ULAB 102-UV UV-Vis spectrophotometer. The ammonia concentration was calculated using a standard curve of ammonium sulfate (NH_4_)_2_SO_4_ solution in μM (ranging from 0.5 to 40 mM).

### Siderophore Production

The siderophore-producing potential of bacterial strains was investigated on Chrome Azurol S agar plates [53]. An aliquot (5 μl) of overnight bacterial culture was placed on the surface of a Chrome azurol-S agar plate. The plates were incubated at 30°C for 7 days, and then observed for the formation of an orange or yellow halo zone around the colonies. The siderophore production index (SPI) was calculated as follows [54]:

SPI = (color conversion area + colony diameter) / colony diameter

### Production of Hydrogen Cyanide (HCN)

Hydrogen cyanide production by the bacterial strains was performed using HCN-sensitive paper [55, 56]. Fifty microliters of bacterial culture were swabbed onto nutrient agar supplemented with filter-sterilized glycine (4.4 g/l). A piece of Whatman paper impregnated with a sodium picrate solution (0.5% picric acid and 2% sodium carbonate) was placed in the lid of a Petri dish. The Petri dishes were then sealed with parafilm and incubated at 30°C for 10 days. HCN production by the isolated strains was indicated by a shift in the paper color from yellow to orange-brown.

### *In Vitro* Antifungal Activity Assay

The antifungal activity of isolated endophytic bacteria against the fungi *Botrytis cinerea* and *Fusarium sporotrichiella* was determined through dual culture assays [57, 58]. A mycelial disk with a diameter of 0.5 cm was cut from PDA plates, where the fungal colony had been previously cultured at 25°C for seven days. The disk was placed in the center of a fresh PDA plate, which was then incubated at 25°C until the colony size reached 3 cm. At a distance of 1.5 cm from the edge of the fungal colony, test bacterial isolates taken from a freshly grown culture were inoculated with an autoclaved toothpick. The control was a Petri dish with a mycelium disc of the corresponding fungus placed in the center on the PDA. The plates were incubated at 25°C. The antifungal activity of the bacteria was assessed once the mycelium in the control dish reached the edge. Treatments were replicated three times. The presence of antifungal activity in bacteria was evidenced by the formation of zones of fungal growth inhibition around the bacterial colony. Colony growth inhibition was calculated by using the following formula [59]:

Inhibition efficiency (%) = (C – T)/C × 100%

where C = Colony growth in control, T = Colony growth in dual culture.

### PGP Assay *in Planta*

**Laboratory Experiment**

Fifty microliters of fresh cultures of the bacterial strains *P. putida* P57, *S. maltophilia* P77, *P. lurida* P88, and P10-1 were transferred to Erlenmeyer flasks containing 50 ml of LB liquid medium and grown in a rotating shaker at 180 rpm for 48 h at 30°C. Bacterial cells were harvested by centrifugation at 3000 g for 30 min at room temperature, washed in sterile 0.9 % NaCl and resuspended in the same solution to a final concentration of 10^7^ CFU/ml. The resulting suspensions were used for Scots pine seed treatment.

*P. sylvestris* L. seeds were surface-sterilized by immersion in 70% ethanol for 1 min, followed by 20 min in 0.1%sodium hypochlorite, and then washed four times with sterile double-distilled water. Seeds (*n* = 100 for each test in triplicate) were treated by soaking separately in 5 ml bacterial suspensions P57, P77, P88, and P10-1 for two hours. Non-inoculated (control) seeds were treated with 5 ml of sterile 0.9 % NaCl. Inoculated and control seeds were sown on filter paper previously moistened with sterile distilled water (control) or 5 ml of bacterial suspensions of tested strains (10^7^ CFU/ml) in Petri dishes to support the colonization process and increase the number of cells adhering to the seeds. The seed germination was conducted under controlled growing conditions (26°C, in darkness for 14 days).

For fourteen-day-old pine seedlings, the following biometric characteristics were assessed: [60]:

● **Total seedling length:** Measured in centimeters using a ruler.

● **Germination percentage (GP):** Calculated for each treatment as a percentage of total germinated seeds.

● **GP** = (number of germinated seeds / numbers of total seeds for bioassay) ×100%

● **Seed vigor index (SVI):** Calculated by multiplying seed germination (%) and seedling length (cm).

● **SVI** = germination percentage (GP) × means of seedling length (cm)

### Field Experiment

The experiment was conducted from May 2022 to June 2024 at the Botanical Garden of the National Forestry University of Ukraine (Lviv, Ukraine). The field plot used in the experiment was 2 m wide and 9 m long. The plot was divided into three sectors based on treatments, which were located 1.5 m apart. The soil texture was sandy loam.

Sterilization of Scots pine seeds and their treatment with bacterial strains P57 or P88 were carried out as described in the 'Laboratory experiment' section. Following treatment, the seeds from both the control and inoculated groups were rinsed with 0.5 liters of sterile distilled water, and excess moisture was subsequently removed using filter paper. Control and inoculated Scots pine seeds were sown in wet soil at a depth of 1-1.5 cm. Three rows of 160 pine seeds each were sown in each sector. The distance between the rows was 50 cm. The percentage of germination for each group of seedlings was assessed on the 35th day after sowing. After counting the germlings in each group, they were watered with the corresponding bacterial suspension (10^7^ CFU/ml) or distilled water for the control group, with a consumption of 0.5 liters of suspension per linear meter. Watering was carried out in moist soil under the seedlings. In the first year of seedling growth, two similar waterings were performed at monthly interval. In the second year of growth, no watering with the tested bacterial cultures was conducted, and the seedlings grew under natural moisturizing. The growth parameters of Scots pine seedlings were measured 5 months and 2 years after sowing. The length of aerial part and root; their fresh and dry weights, and root collar diameter were examined for 10 random seedlings from each row.

### Colonization Assays

**Seed Attachment Assays**

Scots pine seeds were surface-sterilized by immersion in 70% ethanol for 1 min, followed by 20 min in 0.1%sodium hypochlorite, and then washed four times with sterile distilled water. The effectiveness of seed surface sterilization was assessed by placing sterilized seeds on Petri dishes containing LB agar, incubated at 30°C, and checking for bacterial growth after 24 h. Sterilized seeds (*n* = 4 for each test, in triplicate) were treated by soaking separately in 1 ml bacterial suspensions of P57 or P88 (10^7^ CFU/ml) for two hours with gentle agitation at room temperature. Unbound bacteria were removed by sequential washes of the seeds with sufficient 0.9% sterile NaCl. The absence of free bacteria in the washing solution was confirmed by light microscopy [61]. Noninoculated (control) seeds were treated with sterile 0.9% NaCl.

For qualitative adhesion assays, inoculated and control washed seeds were transferred onto filter paper previously moistened with distilled water under aseptic conditions for germination. After the seed coat ruptured and the primary root appeared, the seeds were transferred to Petri dishes containing King's B agar, modified by adding L-glutamine instead of peptone (King's BS agar) [62], supplemented with ampicillin (100 μg/ml), to detect fluorescent *Pseudomonas* bacteria, specifically strains P57 and P88. The Petri dishes were incubated at 30°C for 24 h, after which bacterial growth was checked under UV lighting.

For the quantitative assays, bacterial incubation with the seeds and the washing procedures were performed as described above. One seed was placed in a sterile homogenizer, covered with 1 ml of sterile 0.9% NaCl, and ground until fully homogenized. Serial dilutions of the resulting homogenates were plated (0.05 ml aliquots) on King's BS agar amended with ampicillin (100 μg/ml). The Petri dishes were incubated at 30°C, and the colony-forming units (CFU) of each seed were evaluated after 24 h. Each assay was repeated at least three times.

### Endophytic Colonization Assay

To evaluate endophytic colonization, two randomly selected seedlings from each row, for a total of six per treatment, were harvested two years and five months after sowing (September, 2024). The seedlings were removed from the ground and gently shaken to remove loosely adhering soil from the roots. Seedlings were thoroughly washed under tap water. From each seedling, fragments of the root, stem, and needles were taken, and their surfaces were sterilized by immersion in 1.3% sodium hypochlorite for 5 min, followed by three washes with sterile distilled water [37]. After surface sterilization of the tissue samples (1 g), excess moisture was removed using sterile filter paper. The samples were then cut into approximately 0.05 cm segments using a sterile scalpel. These segments were immersed in Falcon tubes containing 10 ml of sterile 0.9% NaCl and shaken at 28°C and 180 rpm for 30 min [63]. Next, serial dilutions of the resulting suspension were plated (0.05 ml aliquots) on King's BS agar supplemented with ampicillin (100 μg/ml). The Petri dishes were incubated at 30°C, and the CFU for each strain per gram of fresh tissue were evaluated after 7 days.

### Nucleotide Sequence Accession Numbers

The bacterial 16S rRNA gene sequences were deposited in the GenBank nucleotide sequence database under the following accession numbers: OR592462.1 (*Pseudomonas putida* group sp. strain P57), OR647550.1 (*Stenotrophomonas maltophilia* strain P77), OR592463.1 (*Pseudomonas lurida* strain P88), and OR592464.1 (*Pseudomonas lurida* strain P10-1).

### Statistical Analysis

All statistical analyses were conducted using Python with the ‘scipy.stats’ library for performing *t*-tests (version 1.12.0, SciPy Project Team, USA). In all cases, a p-value of ≤ 0.05was considered statistically significant. Pearson correlation analysis was carried out with the ‘NumPy’ library (version 1.26.2). Experiments were performed in triplicate for each variant.

## Results

### Isolation of Endophytic Bacteria from Scots Pine Stem

This study found that Scots pine hosts a variety of culturable endophytic bacteria, with 64 isolates obtained from stem tissues. Among these, 21 Gram-negative isolates underwent further analysis, and four strains—P57, P77, P88, and P10-1—were preliminarily identified as *Pseudomonas* spp. based on phenotypic traits, glucose oxidation/fermentation tests, and catalase activity.

Isolate P57, a fluorescent bacterium, was Gram-negative, rod-shaped, motile, and non-spore-forming, with positive results for catalase, oxidase, and oxidative metabolism, but negative for starch and gelatin hydrolysis, nitrate reduction, and protease. It showed resistance to ampicillin and nalidixic acid.

Isolate P77, a non-fluorescent bacterium, exhibited similar characteristics but was oxidase-negative and protease-positive, with the ability to hydrolyze gelatin. It was resistant to ampicillin and kanamycin.

Isolates P88 and P10-1 had comparable features, being Gram-negative, non-spore-forming rods producing a yellow-green pigment. They tested positive for catalase, oxidase, protease, and gelatinase, but negative for starch hydrolysis and nitrate reduction. Both were resistant to ampicillin, rifampicin, and nalidixic acid.

### 16S rRNA Identification of Bacterial Isolates

**Fig. 1:**
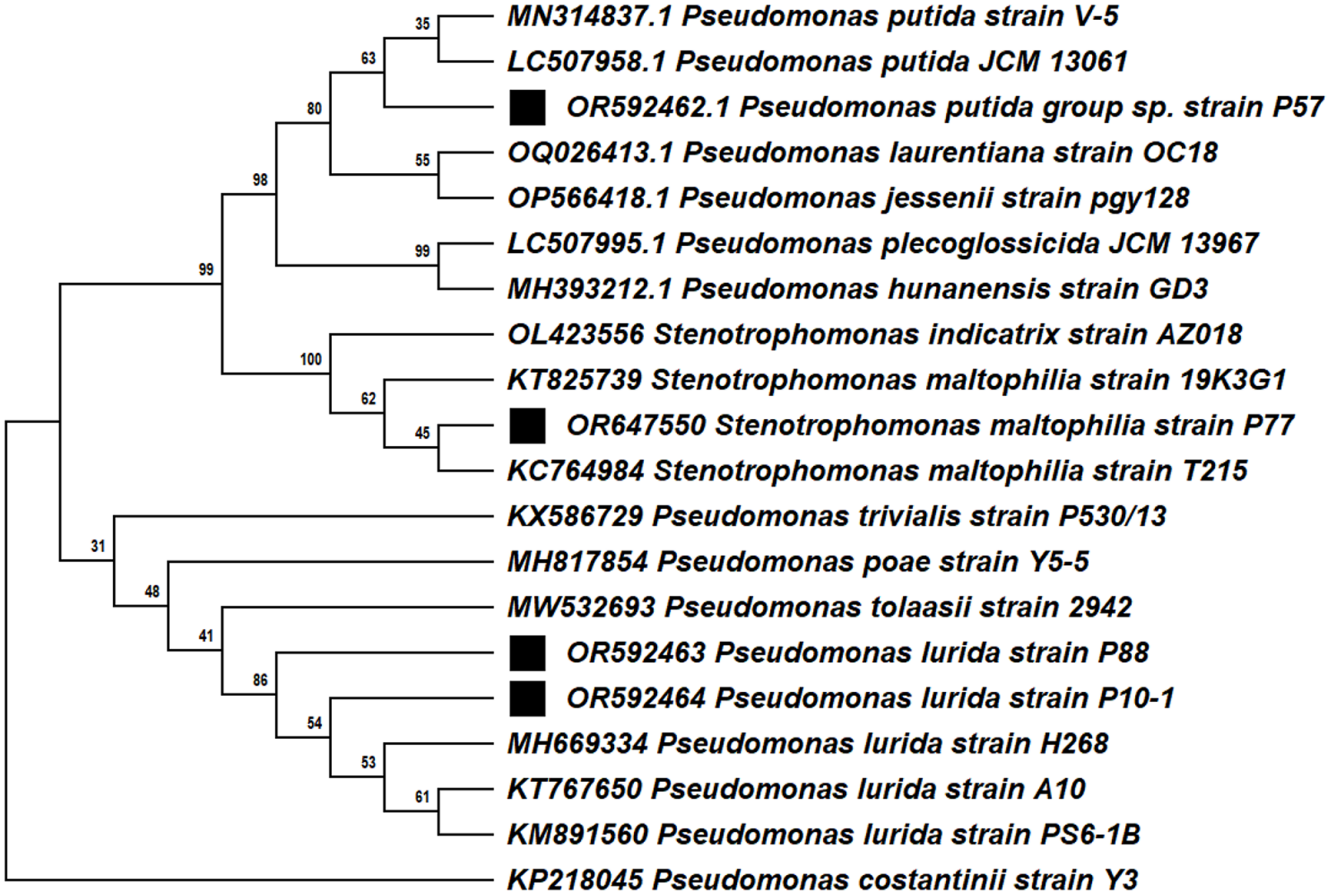
Phylogenetic trees constructed using the maximum likelihood method using MEGA 11 software for the 16S rRNA sequences of bacterial strains. Squares represent bacterial strains in this study.

The species identification of the isolated bacterial strains was determined by sequencing the 16S rRNA gene. The nucleotide sequences of the investigated strains were identified as members of the phylum *Proteobacteria* (*Pseudomonadota*). Three bacterial isolates, P57, P88, and P10-1, belong to the *Pseudomonadaceae* family (genus *Pseudomonas*), while isolate P77 belongs to the *Xanthomonadaceae* family (genus *Stenotrophomonas*). Isolate P57 was identified as *Pseudomonas putida*, showing 99.76% similarity with the reported gene sequence of *P. putida* JCM 13061 (GenBank Accession No. LC507958.1). Isolate P77 exhibited 99.19% similarity with *Stenotrophomonas maltophilia* strain T215 (GenBank Accession No. KC764984). The other strains, named P10-1 and P88, demonstrated 99.78% and 99.50 % similarity with *Pseudomonas lurida* strain H268 (GenBank accession number MH669334), respectively (Fig. 1). Remarkably, this strain was isolated by Liu and coworkers from a pine tree (unpublished).

### PGP Potential of Endophytic Bacteria

The study assessed nitrogen fixation, ammonification, IAA production, phosphate solubilization, siderophore production, and HCN production capabilities of four bacterial strains: *Pseudomonas putida* P57, *Stenotrophomonas maltophilia* P77, and *Pseudomonas lurida* P88 and P10-1.

**Fig. 2:**
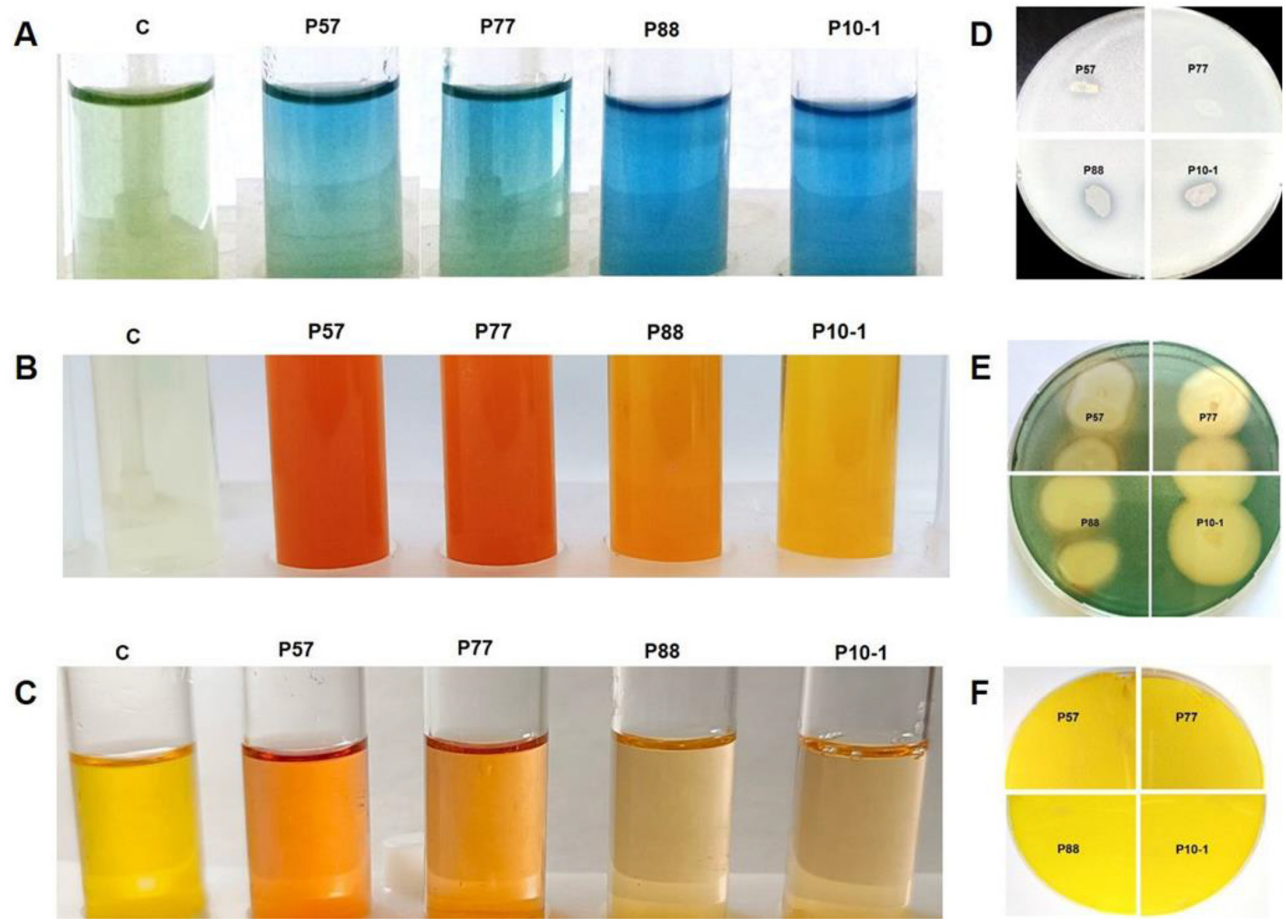
Assessment of the plant growth promotion abilities of the bacterial isolates from Scots pine tissues *P. putida* P57, *S. maltophilia* P77, *P. lurida* P88, and P10-1. (**A**) Nitrogen fixation in NFb medium. (**B**) Ammonia production. (**C**) IAA production. (**D**) Phosphate solubilization. (**E**) Siderophore production. (**F**) HCN production.

*Nitrogen fixation capability.* All strains demonstrated nitrogen-fixing ability in a semi-liquid Nfb-medium (Fig. 2A), indicated by a color shift from green to blue, suggesting they can fix atmospheric nitrogen and alkalize the medium. Additionally, all strains grew on Ashby nitrogen-free medium.

*Ammonification potential.* The strains released ammonia on peptone-rich media (Fig. 2B), with P57 showing the highest level (12.71 ± 0.21 μM/ml), followed by P77 (9.81 ± 0.71 μM/ml), while P88 and P10-1 had lower levels (5.73 ± 0.23 μM/ml and 4.06 ± 0.21 μM/ml, respectively).

*IAA production potential.* All strains produced IAA in the presence of L-tryptophan (Fig. 2C). P57 and P77 exhibited the highest activity (17.74 ± 2.02 μg/ml and 10.41 ± 1.10 μg/ml), while P88 and P10-1 had lower levels (1.24 ± 0.08 μg/ml and 3.78 ± 0.46 μg/ml).

*Phosphate solubilization.* The Pikovskaya test revealed that P57, P88, and P10-1 could solubilize inorganic phosphate, with solubilization indices of 1.11 ± 0.09, 1.47 ± 0.11, and 1.21 ± 0.16, respectively. P77 showed no phosphate solubilization capability (Fig. 2D).

*Siderophore production.* Strains exhibited positive results for siderophore production (Fig. 2E). The plates showed the formation of an orange or yellow halo zone around the colonies. The siderophore production indices for bacterial isolates P57, P77, P88 and P10-1 were 1.33 ± 0.21, 1.27 ± 0.18, 1.44 ± 0.19 and 1.40 ± 0.24, respectively.

*HCN Production.* None of the strains showed the ability to produce HCN (Fig. 2F).

### Antifungal Activity against *B. cinerea* and *F. sporotrichiella*

**Fig. 3:**
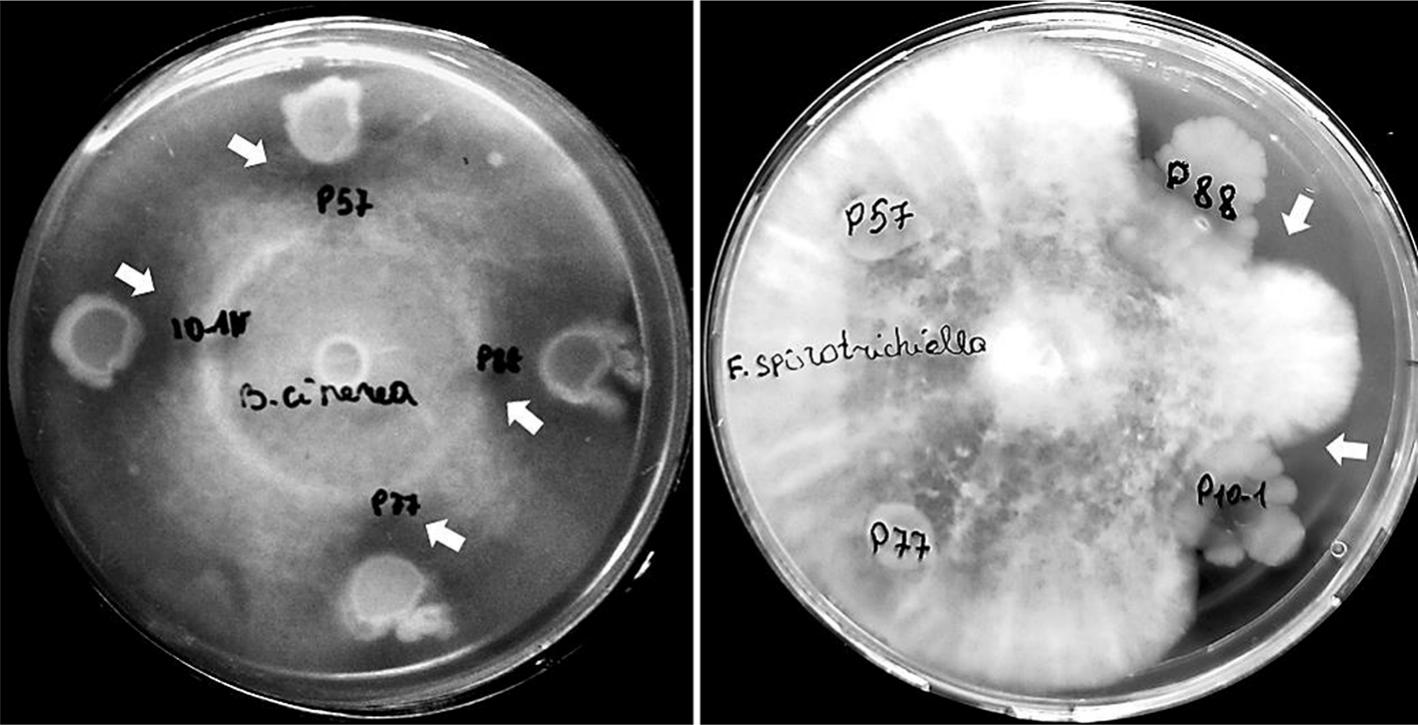
Antifungal activity of the bacterial strains *P. putida* P57, *S. maltophilia* P77, *P. lurida* P88, and P10-1 against *B. cinerea* and *F. sporotrichiella*. The activity was tested using a dual culture assay. Fungal growth inhibition zones are indicated by arrows.

The antifungal activity of *Pseudomonas* strains P57, P88, P10-1, and *Stenotrophomonas* strain P77 was tested on PDA medium against two phytopathogenic fungi: *B. cinerea* and *F. sporotrichiella* (Fig. 3). After 48 h of incubation in dual cultures, we found that all tested bacterial strains exhibited an antagonistic effect against *B. cinerea* which was expressed by the formation of zones of mycelial growth inhibition around bacterial colonies. The percentage of fungal growth inhibition for isolates P57, P77, P88, and P10-1 was 40.3 ± 4.9, 44.2 ± 4.3, 55.6 ± 7.8, and 52.1 ± 6.7, respectively. Notably, only the *P. lurida* strains showed activity against the phytopathogenic fungus *F. sporotrichiella*.

### Effect on Scots Pine Seedling Growth

**Laboratory Experiment**

The comparison between growth parameters of noninoculated and inoculated Scots pine seedlings obtained from the laboratory experiments is presented in Table 1. Pre-sowing treatment of pine seeds with bacterial suspension of the Р88 strain did not have a significant effect GP value compared to control group; the other three strains reduced the number of germinated seeds by 5-8%, depending on the strain. Conversely, seed bacterization increased the total length of seedlings, with a 14% increase observed for strain P57. It also had a positive effect on viability of seedlings (SVI), particularly for strains *P. putida* P57 and *P. lurida* P88.

### Field Experiment

Two bacterial strains of the genus *Pseudomonas*, P57 and P88, which showed the best results in laboratory PGP tests, were used to inoculation of Scots pine seeds that were then sown in the field plot. On the 35th day after sowing, germination rates were assessed. The control group had a germination rate of 46.0 ± 2.0%, while the experimental groups treated with bacterial suspensions P57 and P88 had rates of 71.3 ± 2.8% and 67.7 ± 1.7%, respectively.

**Fig. 4:**
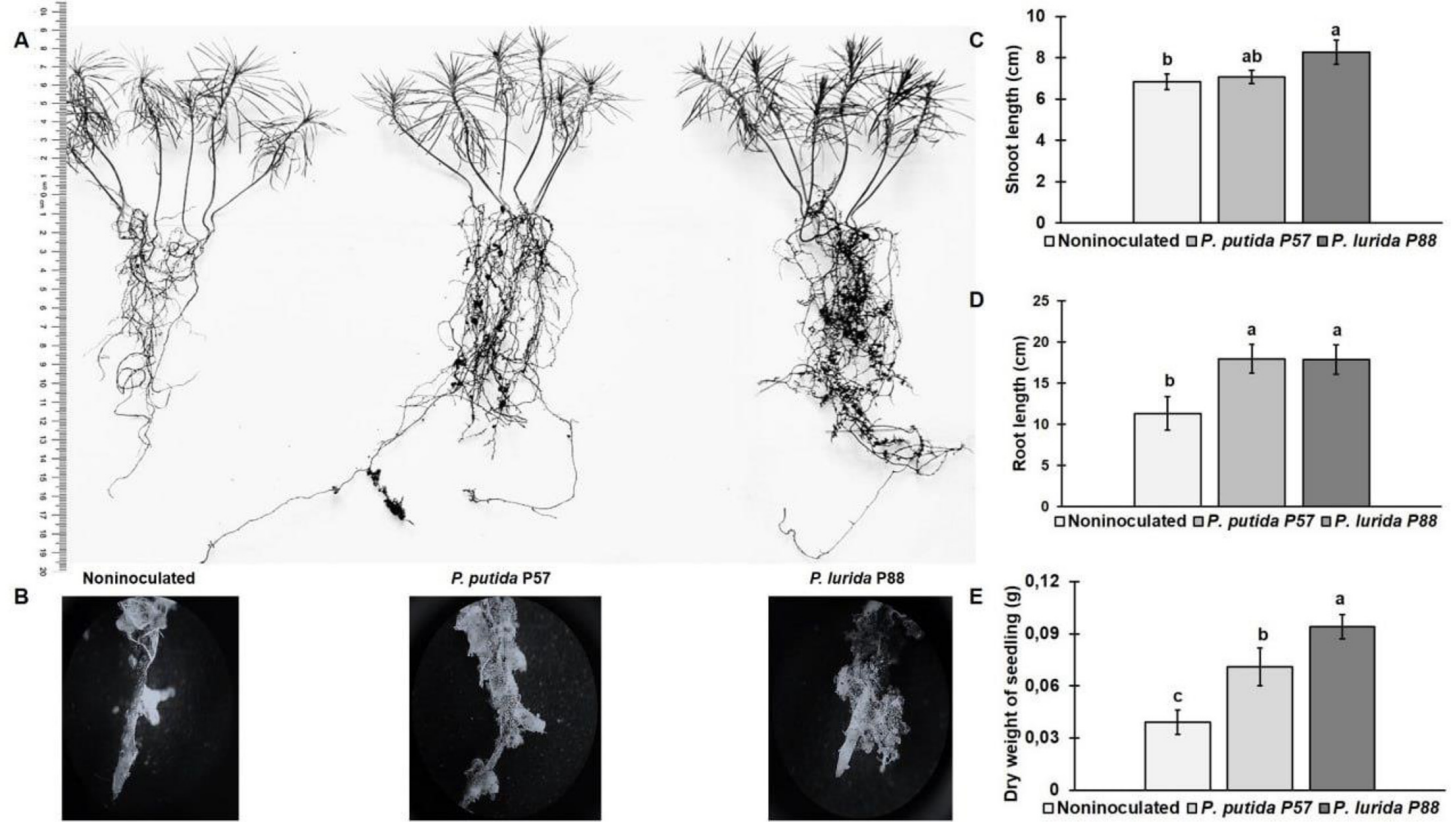
Effect of *P. putida* P57 and *P. lurida* P88 on the morphology (A) and mycorrhization (×20 magnification) (B) of root systems of 5-month-old seedlings and their growth parameters: shoot length (C), root length (D), and dry weights of seedlings (E). Different letters indicate statistically significant differences at *p* ≤ 0.05, *n* = 30. Error bars indicate ± SD.

Visual examination of five-month-old seedlings revealed changes in the morphology of their root systems compared to the control group, including increased root length, branching, enhanced lateral root formation (Fig. 4A) and improved root mycorrhization (Fig. 4B). The root length in inoculated seedlings increased by 58%for both strains compared to the control (Fig. 4D). There were no significant differences in shoot length between the control and P57-inoculated seedlings, while treatment with P88 resulted in a 20.7% increase (Fig. 4C). Bacterial inoculation significantly affected the dry biomass of seedlings with dry weight increasing by 82 % for *P. putida* and by 141% for *P. lurida* (Fig. 4E).

**Fig. 5:**
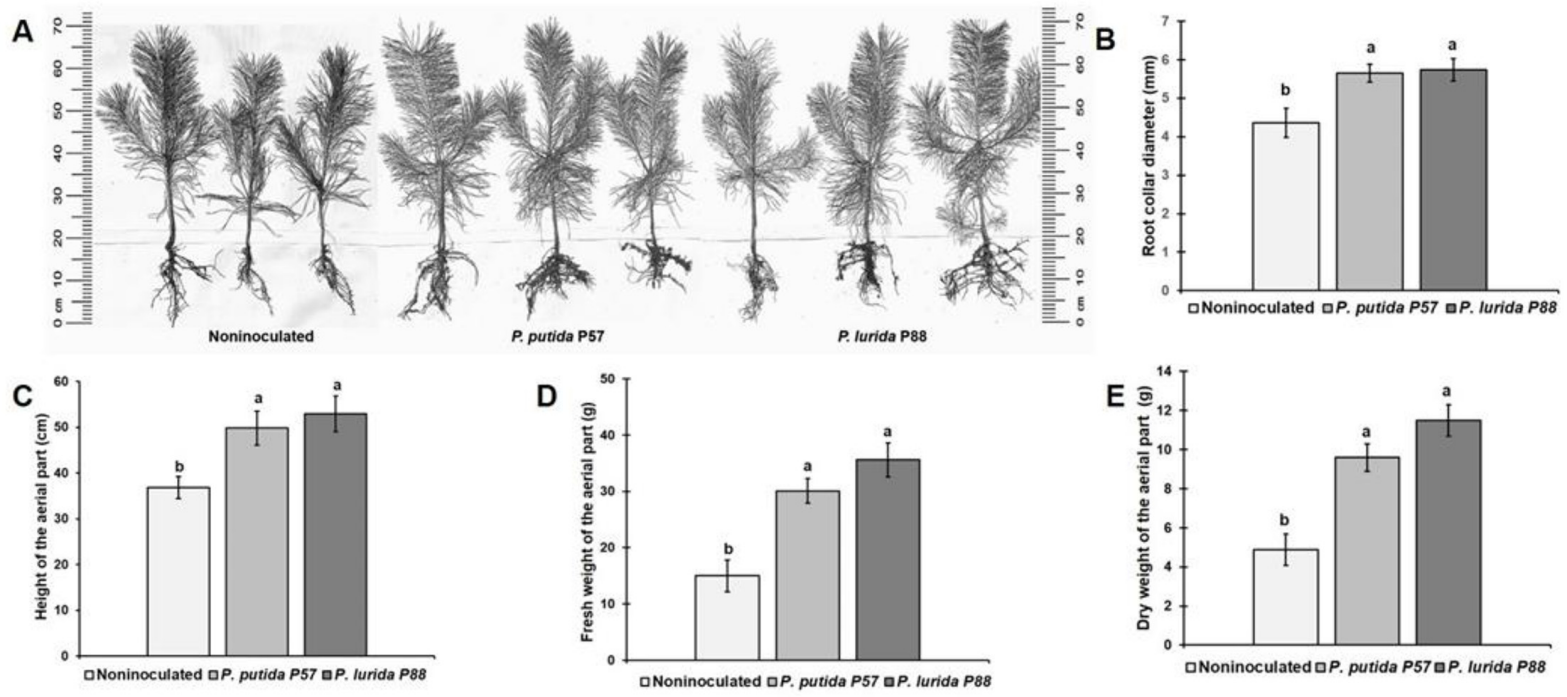
Growth parameters of 2-year-old *P. sylvestris* seedlings inoculated with bacterial suspensions *P. putida* P57 and *P. lurida* P88. (**A**) Noninoculated and inoculated 2-year-old Scots pine seedlings. (**B**) Root collar diameter. (**C**) Aerial part height. (**D, E**) Fresh and dry weight of aerial parts. Bars with the same letter for each compared parameter did not differ significantly at *p* ≤ 0.05, *n* = 30. Error bars indicate ± SD.

The effects of seed inoculation were also assessed in two-year-old Scots pine seedlings grown under field conditions (Fig. 5A). The root system morphology differences seen in five-month-old seedlings persisted in the two-year-old plants (Fig. 5A). Inoculated plants exhibited significant growth parameter increases compared to the control. Specifically, seedlings treated with P57 and P88 were 35.3% and 43.7% taller than the control, respectively (Fig. 5C). The root collar diameter also increased significantly, with P57 and P88 exhibiting increases of 29.6% and 31.4%, respectively (Fig. 5B). The dry weight of the aerial part of inoculated plants was double that of non-inoculated ones, measuring 4.87 ± 0.4 g for control plants, 9.59 ± 0.75 g for *P. putida* P57, and 11.48 ± 0.89 g for *P. lurida* P88 (Fig. 5E).

### Seed and Endophytic Colonization Study

The qualitative and quantitative seed attachment assays indicated adhesion of both bacterial strains, P57 and P88, to the surface of inoculated seeds. As shown in Fig. S1, fluorescent bacteria were primarily localized around the radicle of the germinating inoculated seeds. No growth of fluorescent bacteria was detected around noninoculated seeds. Furthermore, the concentration of P88 bacteria attached to the seed surface was 20 times higher than that of P57 (Table 2). Similarly, a greater number of *P. lurida* colonies, compared to *P. putida*, were found in the internal tissues of the roots and stems of the two-year-old seedlings. Endophytic colonization was not observed in the needles. No evidence of endophytic colonization was found in the control seedlings.

## Discussion

In this study, we isolated endophytic bacteria from the Scots pine stem tissues and tested their growth promotion and antifungal potential *in vitro* and *in planta* trials. Sixty-four bacterial isolates were obtained, with 21 Gram-negative strains selected for further focus on *Pseudomonas* species. We prioritized these bacteria for several reasons: firstly, *Pseudomonas* is one of the dominant endophytic genera in pine, as previously reported [64]; secondly it has been shown to positively influence plant growth through hormone synthesis and improved plant nutrition [65]; thirdly, many species act as biocontrol agents against phytopathogens [66]; and finally, *Pseudomonas* have the ability to mitigate the effects of abiotic stresses [31]. Given Scots pine’s long lifecycle and its exposure to various environmental stresses, the use of *Pseudomonas* strains could be highly beneficial in cultivating resilient planting material for afforestation, particularly in nutrient-poor and disturbed soils.

Four bacterial isolates—P57, P77, P88, and P10-1—exhibited morphological and physiological characteristics typical of the genus *Pseudomonas*. Using 16S rRNA gene sequencing, isolate P57 was identified as *P. putida*, a well-known endophytic bacterium found in various plants, including Scots pine, poplar, pepper, mango, potato, soybean, corn, and rice [22, 67-73].

The isolate P77 was identified as *S. maltophilia*. This bacterium was previously known as *Pseudomonas maltophilia* but was later reclassified into the new genus *Stenotrophomonas*, as *S. maltophilia* [74]. It shares a lot of characteristics with *Pseudomonas* spp., including oxidative and non-fermentative (O+/F-) effects, which are exhibited by only 20% of strains of this species. Therefore, we evaluated the PGP properties of this *Pseudomonas*-like strain alongside the *Pseudomonas* strains isolated in this study. Several studies indicate that *S. maltophilia* has a positive effect on plant growth [75] and serves as an endophytic bacterium in various plants, including pineapple [76], rice [77], safflower [78], and sunflower [79]. Additionally, *S. maltophilia* strain R551-3 has been identified as an endophytic bacterium in the root and stem tissues of poplar [80].

Isolates P88 and P10-1 were identified as *P. lurida*. To our knowledge, this is the first documented isolation of culturable *P. lurida* strains from Scots pine tissues. Previously, this species was isolated from the grass phyllosphere and has also been recognized as an endophyte in poplar [81, 82].

All the strains isolated in this study likely possess plant growth-promoting properties, as similar bacterial species have shown such traits in other plants [83]. Given that strains within the same species can exhibit varied metabolic and functional profiles due to genomic differences [84], studying these new strains offers valuable insights and broadens our understanding of the potential of well-known bacterial species.

The isolates P57, P77, P88, and P10-1 were tested *in vitro* for their PGP activities, with all strains demonstrating at least two significant traits (Table 3). One key PGP mechanism is the production of phytohormones, especially indole-3-acetic acid, which accelerates root growth, and promotes the elongation of roots, resulting in an increased number of root hairs and lateral roots involved in nutrient absorption [85]. All isolates produced IAA only in the presence of L-tryptophan, indicating their use of the L-TRP-dependent pathway. Maximum IAA production was obtained with *P. putida* P57, while *P. lurida* P88 showed the lowest production. IAA production by *Pseudomonas* spp. varies widely, over a range of two orders of magnitude [86]. Such variability is observed not only between different species of this genus but also within the species *P. putida*, even between strains isolated from the same plant species [22]. The two *P. lurida* strains, P88 and P10-1, produced less IAA than *P. putida*, consistent with findings from other studies [87, 88]. The strain *S. maltophilia* P77 synthesized IAA in amounts similar to other plant-associated *Stenotrophomonas* strains, such as *S. maltophilia* JVB5 from the sunflower root endosphere [79], strain Sm97 from wild pistachio trees [89], and *S. maltophilia* BE25 isolated from banana roots [90].

Numerous studies show that endophytic bacteria promote plant growth by enhancing nutrient uptake, including nitrogen, phosphorus, and potassium [91]. Atmospheric nitrogen (N_2_) is inaccessible to most organisms, but diazotrophic bacteria convert it through biological nitrogen fixation (BNF) [92, 93]. In our study, all isolates—P57, P77, P88, and P10-1—grew on nitrogen-free media (Ashby and Nfb), suggesting nitrogen-fixing capabilities. The highest potential for nitrogen fixation on Nfb semi-solid medium was demonstrated by *P. lurida* strains, followed by *S. maltophilia*, with *P. putida* showing the lowest potential (Fig. 2A). *Pseudomonas* is known for nitrogen fixation [94], though *P. putida*'s ability remains debated [95]. However, Li *et al*. [96] identified nitrogen-fixing strains (AY1 and CN15) from the sugarcane rhizosphere that showed positive results in the acetylene reduction assay and expressed the *nifH* gene. *P. putida* TS 18, isolated from *Spathodea campanulata*, also exhibited nitrogen fixing potential [97]. As for another representative of the genus *Pseudomonas*, *P. lurida*, the EOO26 strain has been reported to fix nitrogen under salt stress [98]. Several studies have indicated N_2_-fixing properties in different strains of *S. maltophilia*: S255 from rhizosphere [99], C6 from chickpea nodules [100], MAGDE3 and MANC from cassava [101]. This evidence highlights the potential of these isolates to contribute to plant growth by improving nitrogen availability.

Ammonia production is an important characteristic of the PGP trait that influences growth and can aid in biocontrol against pathogens. In the current study, all four strains showed the ability to convert organic nitrogen from peptone into ammonia, as illustrated in Fig. 2B. Notably, *P. lurida* strains P88 and P10-1, which had high nitrogen fixation potential on semi-solid Nbf medium, produced less ammonia on peptone-rich medium compared to *P. putida* P57 and *S. maltophilia* P77, which were weaker nitrogen fixers. High ammonia production was also observed in *P. putida* PS8 from the roots of *P. sylvestris* [22]. The ability to convert organic nitrogen into ammonia was also observed in *P. lurida* strains: EOO26 from the roots of *Odontarrhena obovata* [98] and AH1 from the rhizosphere of *Aconitum heterophyllum* [87]. *S. maltophilia* strains have also shown ammonia production traits [100, 102].

Phosphorus is one of the most important nutrient elements for plants. Despite the presence of phosphorus compounds in agricultural soils, most exist in an insoluble form [103]. Among the tested strains, only *Pseudomonas* strains exhibited phosphate-solubilizing properties, as previously demonstrated for strains PS8 from *P. sylvestris* [22] and AF8 from wild poplar stems [82]. The negative result in phosphate solubilization for strain P77 was unexpected, as it was previously reported that *S. maltophilia* JVB5 was a strong mineral phosphate solubilizers [79].

Siderophore production is an important characteristic of plant-associated microorganisms, indicating their ability to improve the bioavailability of iron to plants through their chelating properties, thereby positively influencing the growth of the host organism. In this study, all four bacterial strains exhibited siderophore production. The fluorescent strains P57, P88, and P10-1 likely synthesize pyoverdine, a known siderophore of *Pseudomonas* [104], while strain P77 appears to produce catechol-type siderophores typical of *Stenotrophomonas* [105]. Previous studies have shown that bacterial siderophores can inhibit phytopathogenic mold growth, with antifungal activity linked to siderophore concentration [106]. By sequestering iron and limiting its availability to pathogens, siderophore secretion serves as an effective biocontrol mechanism used by bacteria to suppress pathogen growth [13, 65].

In addition to siderophores, endophytic bacteria can antagonize phytopathogens by producing hydrogen cyanide. In the current study, none of the isolates produced HCN. However, HCN absence did not affect their biocontrol abilities, as they utilized other pathogen inhibition mechanisms [89]. In dual culture tests, all strains inhibited the growth of the phytopathogen *B. cinerea*, with inhibition zones strongly correlated with siderophore production (r = 0.84). Additionally, only *P. lurida* strains suppressed the mycelium growth of *F. sporotrichiella*, suggesting they may secrete other antifungal compounds besides siderophores. Thus, multiple mechanisms contribute to the antifungal activity of these bacteria.

The PGP abilities of isolates P57, P77, P88, and P10-1 *in vivo* were evaluated by a Scots pine seed germination test on Petri dishes. The test also assessed the pathogenicity of these strains to the natural host seedlings. We have previously established the pathogenicity of several *Bacillus pumilus* strains isolated from Scots pine tissues to host seedlings in seed germination tests [107]. However, the good biocontrol potential of this bacterium against phytopathogenic fungi has also been reported [108, 109]. In this study, none of the tested strains caused visible seedling pathologies. A slight reduction in germination (5–8%) was observed for all strains, likely due to the production of auxin, which can disrupt the gibberellin/abscisic acid balance, key regulators of seed germination [110]. Additionally, bacteria entering seeds could compete with the embryo for nutrients during germination. Despite the germination reduction, inoculation positively affected seedling length, especially with *P. putida* P57, the most active auxin producer among the strains.

The germination experiment results were validated in a two-year field trial using two strains, P57 and P88, which showed the best PGP results *in vitro* and *in vivo* (Tables 1 and 3). Due to *Stenotrophomonas maltophilia*'s pathogenic potential in humans, it was excluded from field testing [111].

Inoculation outcomes were assessed at three stages: germination rate on the 35th day after sowing, growth parameters after five months, and two years later. Unlike lab results, field trials showed increased seedling numbers with strain P57 (54% increase) and P88 (41% increase). Seed germination under natural conditions is one of the most vulnerable stages in plant ontogenesis. Once the seed coat is disrupted, the germinating embryo encounters soil microbiota, including soil-borne pathogens. It can be assumed that the improved germination of inoculated seedlings may be attributed to the biocontrol properties of these bacteria, as demonstrated in this study. Previous research has highlighted the biocontrol activity of *Pseudomonas putida* strains against several pathogens, including *Xanthomonas fragariae* in strawberries [112], *Fusarium oxysporum* in tomatoes [113], and others like *Rhizoctonia solani* and *Pectobacterium atrosepticum* [114]. Similarly, *P. lurida* AH1 has shown antagonism against various phytopathogenic fungi [87].

Bacterial inoculation significantly improved root system development of five-month-old Scots pine seedlings compared to noninoculated seedlings, resulting in longer and more branched roots for both strains (Fig. 4). However, while no significant shoot length difference was found between seedlings inoculated with P57 and control, inoculation with P88 increased shoot length by 20.7%. The treatment also enhanced biomass of inoculated seedlings due to the more developed root systems. This effect is likely attributed to the influence of exogenous IAA, which stimulates the formation of lateral and adventitious roots [115]. Similarly, *Pseudomonas abietaniphila* BHJ04, an IAA-producing strain, was found to significantly promote root and branch growth in *Pinus massoniana* [63]. Despite the bacterial inoculation, Scots pine seedlings, known for requiring mycorrhizal fungi for optimal growth, showed high levels of mycorrhization in both treated and untreated groups (Fig. 4B).

Inoculation with *P. putida* and *P. lurida* had a positive impact on the growth parameters of two-year-old seedlings, exceeding the control group in height, root collar diameter, and above-ground biomass. This growth boost was likely due to the vigorous root system development observed in five-month-old seedlings, where increased lateral roots resulted in a more branched system for nutrient absorption. The positive effects of *P. putida* on plant growth have been widely documented. For example, *P. putida* PS8 improved the biometric parameters and dry weight of sunflower seedlings [22], and the strain KT2440 enhanced growth in maize seedlings [116]. Similarly, *P. putida* UW4 promoted root and shoot development in *Pinus pinaster* [117]. Although reports on *P. lurida*'s PGP properties are limited, strain EOO26 was found to stimulate sunflower growth [98].

Endophytic colonization appears to be a key factor in growth promotion. Greenhouse trials show that the size of the bacterial population directly correlates with the effectiveness of growth enhancement in conifers like spruce, lodgepole pine, and Chinese red pine [24, 37, 63]. Puri *et al*. [37] noted that the best colonizers in trials increased seedling length by up to 60% and biomass by up to 302%.

The initial phase of colonization involves bacterial adhesion to the seed, which is essential for root colonization [118, 119]. In a quantitative seed attachment assay, both tested strains adhered to the seed coat, with *Pseudomonas lurida* showing greater effectiveness (Table 2). Studies indicate that *P. putida* has structural elements like adhesins and exopolysaccharides that aid in attachment to seeds and roots [95]. In a qualitative test (Fig. S1), after the seed coat was disrupted, bacteria concentrated around the emerging radicle, attracted by root exudates such as sugars, organic acids, amino acids, and flavonoids, which serve as nutrients. This active chemotaxis toward root exudates is crucial for successful plant root colonization [95].

The endophytic population sizes of strains P57 and P88 in the roots and stems were significantly lower (tens to hundreds of times) than those reported for other PGP bacteria in inoculated conifers under controlled conditions [37, 63, 120]. This is likely due to natural field conditions with various biotic and abiotic stresses. Shishido and Chanway [121] observed a 10- to 100-fold reduction in PGPR populations in spruce seedlings following a transition from greenhouse to field conditions, citing the harsher field environment as the cause. Despite reduced population sizes, significant growth promotion (up to 234%) was noted in spruce.

In this study, two-year-old pine seedlings inoculated with *Pseudomonas* strains and grown entirely under field conditions showed an increase in biomass of the aerial part by 80-140% despite the low level of colonization in the roots and stems (10^2^-10^3^ CFU/g fresh tissue). This supports the idea that no specific population size threshold is needed for endophytic PGP bacteria to promote growth, as they can be effective even at low numbers by occupying optimal niches within the host [121].

Detection of *P. putida* P57 and *P. lurida* P88 in the internal tissues at the end of the growing season indicates the formation of stable endophytic colonies under field conditions, essential for their beneficial effects.

## Conclusion

The findings indicate that *P. putida* and *P. lurida* strains, isolated from Scots pine, have the potential to significantly enhance the host plant's growth. *In vitro* tests demonstrated their strong PGP capabilities, including nitrogen fixation, phosphate solubilization, siderophore, IAA, and ammonia production, which are vital for improving plant yield. These strains effectively colonize pine seedling tissues, positively influencing plant development through their functional traits.

This research represents one of the few long-term studies to successfully apply endophytic *Pseudomonas* strains for field cultivation of natural host seedlings. The results suggest that these strains, with their varied PGP properties, could serve as promising bioinoculants for Scots pine cultivation, boosting biotic resilience in diverse soil conditions amid global climate change.

## Supplemental Materials

Supplementary data for this paper are available on-line only at http://jmb.or.kr.



## Figures and Tables

**Table 1:** Effect inoculation of endophytic bacteria from Scots pine on growth parameters of 14-day-old *P. sylvestris* (L.) seedlings (laboratory experiment).

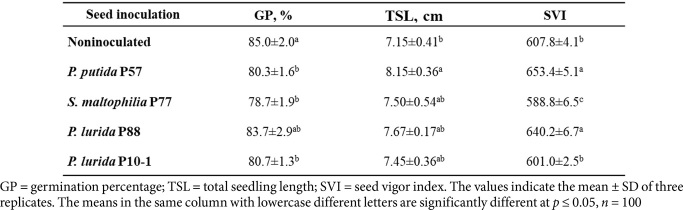

**Table 2:** Colonization of seeds and Scots pine tissues under inoculation with *Pseudomonas* strains.

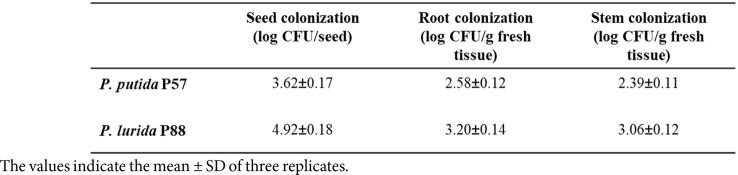

**Table 3:** Plant growth-promoting features of endophytic bacterial strains from Scots pine.