# Supplementary material for: Isolation and Characterization of Multi-Trait Plant Growth-Promoting Endophytic Bacteria from Scots Pine Tissues
Source: J Microbiol Biotechnol. 2024 Nov 22;35:e2408056. doi: 10.4014/jmb.2408.08056 (PMC11813345; doi:10.4014/jmb.2408.08056)
Supplement: Supplementary file 1 [file jmb-35-e2408056-supple.pdf]

Supplementary Figure

Isolation and characterization of multi-trait plant growth-promoting endophytic bacteria from Scots pine tissues

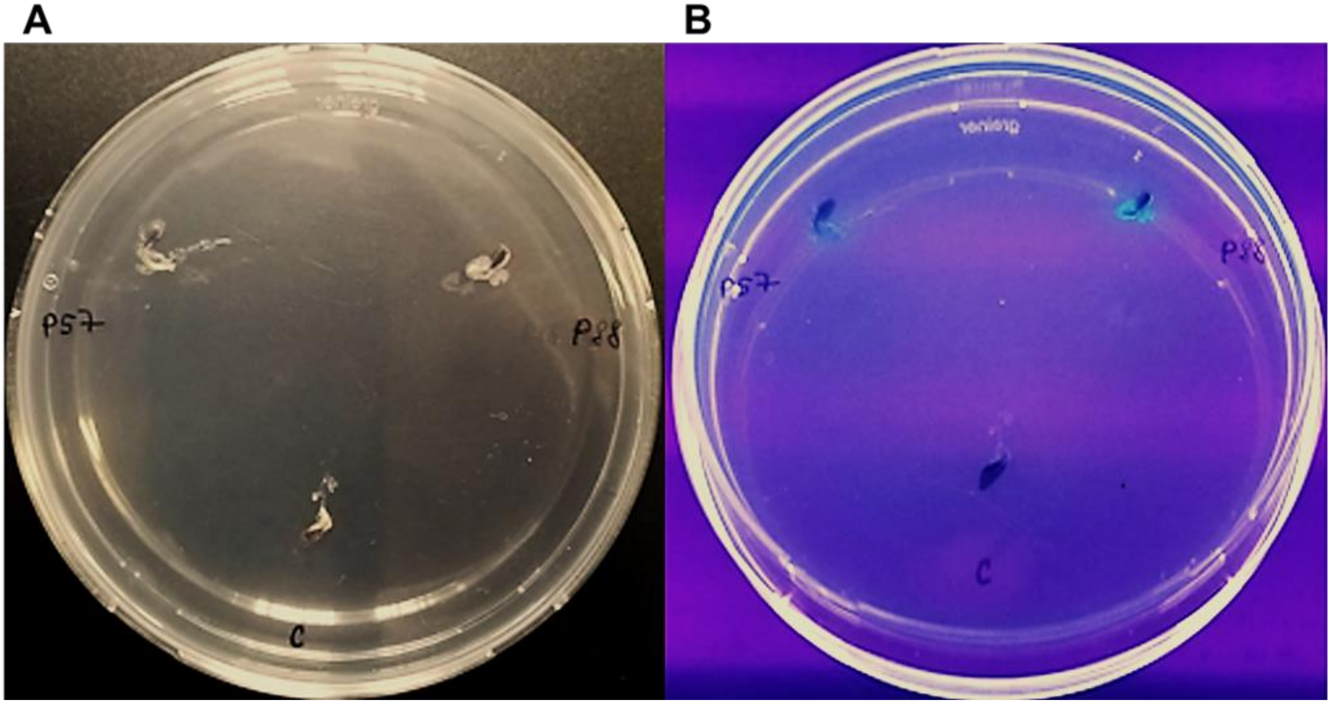

Fig. S1 Seed attachment assay. Under sterile conditions, Scots pine seeds inoculated with P57 or P88 strains, as well as controls, were germinated on filter paper moistened with distilled water. When the primary root emerged through the broken seed coat, the seeds were transferred to Petri dishes containing King's BS agar and ampicillin (100 μg/mL). Incubation at 30°C for 24 hours followed, after which bacterial growth was assessed by visual observation (**A**) and UV examination (**B**).
